# Large language models in cancer: potentials, risks, and safeguards

**DOI:** 10.1093/bjrai/ubae019

**Published:** 2024-12-20

**Authors:** Md Muntasir Zitu, Tuan Dung Le, Thanh Duong, Shohreh Haddadan, Melany Garcia, Rossybelle Amorrortu, Yayi Zhao, Dana E Rollison, Thanh Thieu

**Affiliations:** Department of Cancer Epidemiology, Moffitt Cancer Center, Tampa, FL, Moffitt Cancer Center and Research Institute, Tampa, FL 33612, United States; Department of Machine Learning, Moffitt Cancer Center, Tampa, FL, Moffitt Cancer Center and Research Institute, Tampa, FL 33612, United States; Department of Machine Learning, Moffitt Cancer Center, Tampa, FL, Moffitt Cancer Center and Research Institute, Tampa, FL 33612, United States; Department of Machine Learning, Moffitt Cancer Center, Tampa, FL, Moffitt Cancer Center and Research Institute, Tampa, FL 33612, United States; Department of Machine Learning, Moffitt Cancer Center, Tampa, FL, Moffitt Cancer Center and Research Institute, Tampa, FL 33612, United States; Department of Cancer Epidemiology, Moffitt Cancer Center, Tampa, FL, Moffitt Cancer Center and Research Institute, Tampa, FL 33612, United States; Department of Cancer Epidemiology, Moffitt Cancer Center, Tampa, FL, Moffitt Cancer Center and Research Institute, Tampa, FL 33612, United States; Department of Cancer Epidemiology, Moffitt Cancer Center, Tampa, FL, Moffitt Cancer Center and Research Institute, Tampa, FL 33612, United States; Department of Cancer Epidemiology, Moffitt Cancer Center, Tampa, FL, Moffitt Cancer Center and Research Institute, Tampa, FL 33612, United States; Department of Machine Learning, Moffitt Cancer Center, Tampa, FL, Moffitt Cancer Center and Research Institute, Tampa, FL 33612, United States

**Keywords:** large language models, natural language processing, ChatGPT, cancer, artificial intelligence, potentials, risks, safeguards, chatbots

## Abstract

This review examines the use of large language models (LLMs) in cancer, analysing articles sourced from PubMed, Embase, and Ovid Medline, published between 2017 and 2024. Our search strategy included terms related to LLMs, cancer research, risks, safeguards, and ethical issues, focusing on studies that utilized text-based data. 59 articles were included in the review, categorized into 3 segments: quantitative studies on LLMs, chatbot-focused studies, and qualitative discussions on LLMs on cancer. Quantitative studies highlight LLMs’ advanced capabilities in natural language processing (NLP), while chatbot-focused articles demonstrate their potential in clinical support and data management. Qualitative research underscores the broader implications of LLMs, including the risks and ethical considerations. Our findings suggest that LLMs, notably ChatGPT, have potential in data analysis, patient interaction, and personalized treatment in cancer care. However, the review identifies critical risks, including data biases and ethical challenges. We emphasize the need for regulatory oversight, targeted model development, and continuous evaluation. In conclusion, integrating LLMs in cancer research offers promising prospects but necessitates a balanced approach focusing on accuracy, ethical integrity, and data privacy. This review underscores the need for further study, encouraging responsible exploration and application of artificial intelligence in oncology.

## Introduction 

Cancer remains a global health threat, with high mortality rates and increasing incidence rates.^1–3^ From treatment to survivorship, cancer care involves complex decision-making, requiring the integration of vast amounts of relevant data and germane knowledge.^2^^,^^4^^,^^5^ Despite progress, translating medical data into actionable insights remains a challenge.^6^^,^^7^ In recent decades, there has been a concerted effort to harness the potential of artificial intelligence (AI) in medicine.^8^^,^^9^ The emergence of advanced computational methods, especially large language models (LLMs), offers new possibilities for improvement and change.^10^^,^^11^ With their capability of data analysis, patient communication, and quickening the pace of discovery, LLMs hold promise to influence the approach to cancer research and patient care.^11^^,^^12^

Initially, language models struggled with processing medical texts due to the scale and complexity of the data.^13^^,^^14^ However, advancements in AI have enabled contemporary state-of-the-art models like GPT (generative pretrained transformer), Claude 2, Llama 3, and PaLM to handle large datasets and generate insights with greater nuance and accuracy.^15–17^ These models, built on deep learning architectures and trained on vast datasets, excel in various natural language processing (NLP) tasks, such as summarization, translation, sentiment analysis, and text generation.^10^^,15-^^17^ Furthermore, innovations such as the attention mechanism, significant increases in model size, and access to diverse training data have been critical in enabling these models to excel in complex tasks.^18^ The attention mechanism, in particular, allows models to focus on relevant parts of the input, while larger model sizes and extensive training data enhance performance and generalization across NLP tasks.^10^^,^^15–17^ Despite these advancements, the integration of LLMs into oncology practice remains limited. Factors such as the need for specialized oncology training, concerns about data privacy, regulatory and ethical issues, and challenges in integration with existing healthcare systems might contribute to this gap, highlighting the complexity of applying advanced AI in high-stake medical fields.^19^

Despite these challenges, AI adoption in oncology is growing, particularly with tools like ChatGPT and specialized chatbots. The integration of these technologies into cancer research and patient care reflects a shift in AI’s role in medical science.^20–22^ These tools, especially LLMs, demonstrate AI’s increasing feasibility and utility, enabling researchers to discover patterns and correlations within vast medical repositories such as scientific literature, patient medical records, and clinical trial data.^23^ This adaptation has supported oncologists and researchers to interpret complex medical information, predict patient outcomes, and even assist in developing personalized treatment plans.^24^^,^^25^

Amid the global cancer incidence and mortality rates, there is a need to integrate vast medical data for informed clinical decisions in cancer care.^1^^,^^3^^,^^26^ While advanced computational methods, particularly LLMs, hold a transformative potential in oncology, there is a gap in synthesizing these developments. This review seeks to bridge this gap by providing an overview of LLM applications in cancer research, highlighting technological progress, practical applications, and theoretical perspectives. We specifically sought to profile the existing literature by outlining the potentials, risks, and safeguards of LLM, categorizing relevant studies into 3 domains: (a) quantitative methods of LLMs, consisting of studies applying LLMs to data analysis, NLP tasks, and predictive modelling with measurable outcomes; (b) chatbot-focused studies, focused on LLM-powered chatbots for patient interactions, treatment support, and clinical decision-making; and (c) qualitative discussions on LLMs’ broader impacts on oncology, exploring ethical issues, risks, and societal impacts of LLMs. This approach highlights the relevance of LLMs in cancer care and research, exploring their potential, risks, and safeguards. Readers are encouraged to consult Table 1 for definitions of key terms used throughout the article.

**Table 1.** Glossary of key terms.

Term	Definition
Artificial intelligence (AI)	The development of systems that can perform tasks requiring human-like intelligence, such as learning and problem-solving.
Natural language processing (NLP)	Enabling machines to understand and process human language.
Large language models (LLMs)	Advanced AI models for understanding, interpreting, and generating human language.
Named entity recognition (NER)	An NLP task that identifies and classifies named entities in text into categories.
BiLSTM (bidirectional long short-term memory)	A recurrent neural network that processes data in both forward and backward directions.
Attention mechanism	A technique allowing models to focus on specific parts of input data.
Transformer models	Deep learning models using self-attention mechanisms.
BERT (bidirectional encoder representations from transformers)	A transformer-based machine learning technique designed to better understand the context of words in search queries.
GPT (generative pretrained transformer)	LLM for generating human-like text.
ChatGPT	A GPT variant optimized for conversational applications.
Chatbot	Software simulating human-like conversation using AI and NLP.
Embedding	Converting words or phrases into vectors to represent their meaning.
Zero-shot learning	The ability of a model to understand and respond to tasks it has not been specifically trained on.
Encoder	Processes input data into a richer representation in neural networks.
Token	The basic unit of text processing in NLP.
Tokenization	The process of converting text into tokens can be fed into NLP models.
Fine-tuning	Adjusting a pre-trained model for a specific task.
Prompt engineering	Creating inputs to elicit specific responses from LLMs.
Precision	The measure of a model’s performance in correctly identifying only relevant instances
Recall	Measuring a model’s performance in capturing all relevant instances.
F1 score	A measure of a test’s accuracy, considering precision and recall.
AUC (area under the curve)	A performance metric for classification models at various threshold settings.
Macro-F1 score	A type of F1 score calculated by taking the average of the F1 scores per class, giving equal weight to each class.
Micro-F1 score	An F1 score considering the total true positives, false negatives, and false positives.
Cross-validation	A technique for assessing how a predictive model will generalize to an independent dataset.
ROC (receiver operating characteristic) curve	A graph showing the performance of a classification model at all classification thresholds.
Physical component summary (PCS)	A health survey score reflects a person’s physical well-being and ability to perform everyday activities.

This table provides a comprehensive glossary of key terms and acronyms used in the field of AI, with a focus on NLP and LLMs.

## Methods

### Scope and purpose

Our literature review explores the diverse applications and implications of LLMs in oncology. Our methodology adopts a narrative review approach influenced by the Preferred Reporting Items for Systematic Reviews and Meta-Analyses (PRISMA) framework.^27–30^ Our method differs from systematic reviews, which rigorously identify all pertinent research articles following a strict guideline.^28–30^ We sought to provide an overview of current knowledge and trends in LLMs within cancer research, addressing technological advancements, practical applications, and theoretical perspectives. This review aims to synthesize insights across various studies to better understand the potential, risks, and safeguards of LLMs in cancer research.

**Figure 1.**
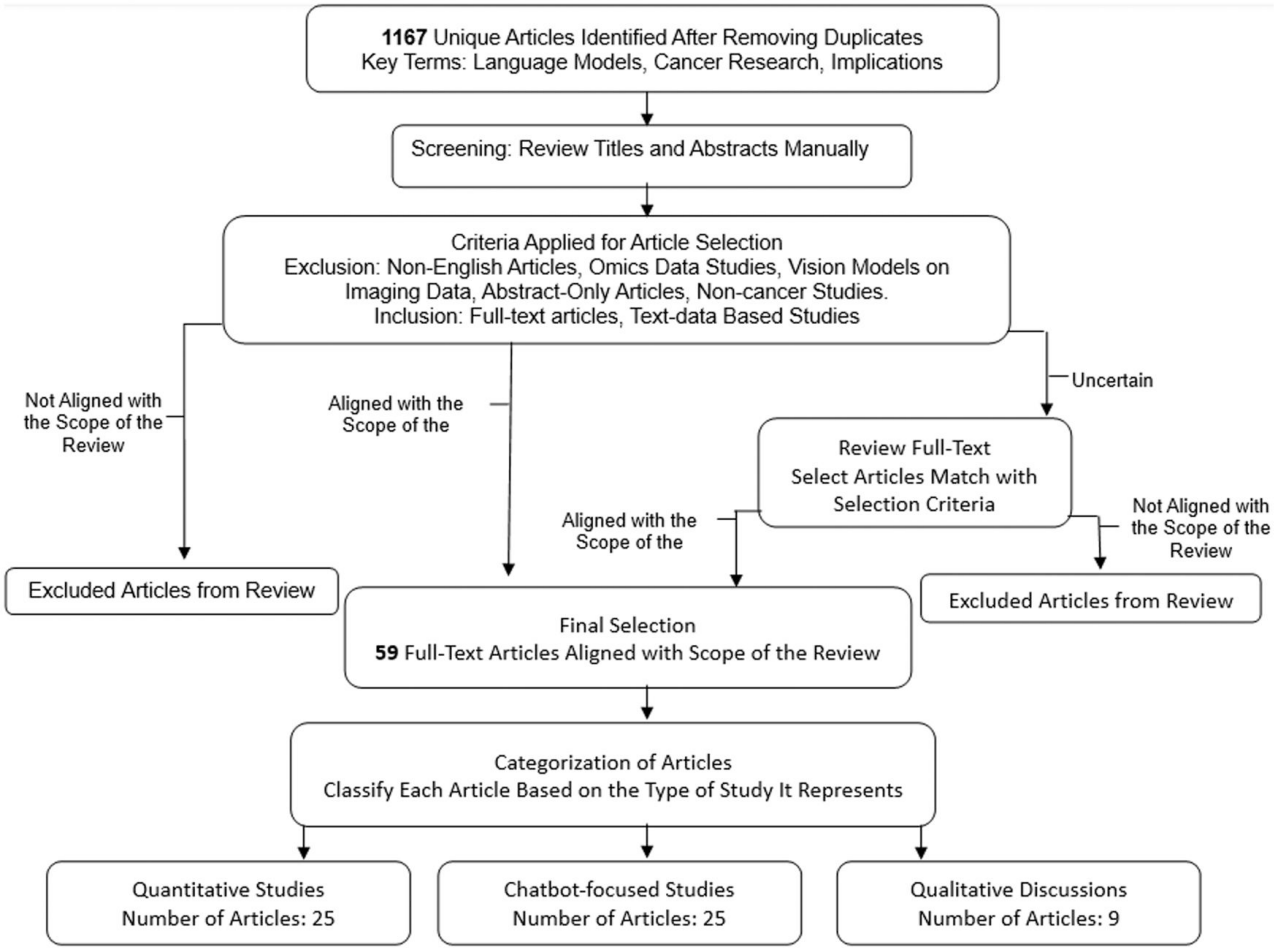
Literature review process for LLMs in oncology. Flowchart depicting the article selection process for our review on LLMs in oncology. Initially, 1167 articles were identified through a database search. Each article was manually assessed by title and abstract for relevance to the review scope. Articles were classified as “aligned with scope of the review”, “not aligned with scope of the review”, or “uncertain”. For articles marked as “uncertain”, a full-text review was conducted to determine their relevance to our study. Finally, our process led us to a final selection of 59 relevant articles aligned with the scope of the review. LLMs = large language models.

As depicted in Figure 1, the review process follows a structured approach beginning with a search across multiple databases, followed by a screening and selection methodology, ultimately categorizing selected articles into quantitative studies, chatbot-focused studies, and qualitative studies.

### Eligibility criteria

We included full-text articles that focused on LLM applications in text-based data relevant to cancer research. Studies such as vision-based models on imaging data, omics data,^31^ and those unrelated to cancer were excluded.

### Literature search strategy

We conducted a search across 3 databases: PubMed, Embase, and Ovid Medline, covering a range of article types, including journal and conference articles. Our strategy involved key terms related to 3 main themes: language models (“large language models”, “BERT”, “GPT”, “transformers”), cancer research (“cancer”, “oncology”, “tumor”, “carcinoma”, “malignancy”, “neoplasm”, “immunotherapy”, “chemotherapy”, “radiation therapy”), and associated implications (“risks”, “safeguards”, “potentials”, “challenges”, “limitations”, “threats”, “concerns”, “ethical issues”, “security issues”). These terms were combined using “and” and “or” conditions and searched within titles and abstracts. The search covered publications from 2017 to September 2024 and was limited to English-language articles.

### Study selection

Five reviewers conducted the review. Two reviewers developed the selection criteria and search terms for the study and then performed initial screening to identify relevant articles. They manually reviewed articles that aligned with the scope of the review, narrowing the list from the 1167 initially identified to the final selection. Upon manual review of the title and abstract section, the identified 1167 articles were classified as “aligned with scope of the review” if they matched the eligibility criteria, “not aligned with scope of the review” if they were clearly outside the eligibility criteria, or “uncertain” if their relevance was not apparent from the abstract and title alone. Articles recognized as “aligned with scope of the review” were retained for the final full-text review. In contrast, those “not aligned with scope of the review” were excluded from subsequent analysis. The “uncertain” articles underwent a full-text review to assess their content against our eligibility criteria. Ultimately, the process led us to a final selection of 59 relevant articles aligned with the scope of our review.

All 5 reviewers participated in the detailed manual review, with each reviewer independently assessing assigned articles. Regular meetings were held to discuss findings and address challenges, ensuring a rigorous review.

## Results

### Summary of selected studies

A total of 59 full-text articles were included in our study. The articles were categorized into 3 distinct groups: 25 focused on quantitative methods of LLMs (see Table 2), 25 chatbot-focused studies (see Table 3), and 9 qualitative discussions on LLMs’ broader impacts on oncology (see Table 4).

**Table 2.** Overview of quantitative studies utilizing LLM in cancer research.

Author and year	LLM model	Data types and sources	NLP task formulation with aim	Sample size (training/validation/test)	Performance metrics
Santos et al, 2022^32^	PathologyBERT (transformer model based on BERT, traditional 12-layer architecture)	Histopathology specimen reports from Emory University Hospital	Masked Language Prediction aimed at classifying breast cancer diagnosis	347 173 reports Training: 238 342, Validation: 34 050, Test: 68 100; Breast cancer diagnosis classification: not specified	15% masked prediction overall accuracy: 0.73, Top 5 accuracy: 0.83; Breast cancer diagnosis classification: F1 score: 0.70 (specific for non-breast cancer label)
Zhang et al, 2021^33^	BERT-based BiLSTM-Transformer network (BERT-BTN) with pre-training. Uses BERT-WWM for embedding, BiLSTM for sentence structure, and transformer layer for global dependencies.	Chinese chest CT reports from Peking University Cancer Hospital	NER aimed at enhancing lung cancer screening and staging by extracting key clinical entities	359 reports Training: 70%, Validation: 10%, Test: 20%.	Under the exact match scheme, macro-F1 score: 85.96%; Micro-F1 score: 90.67%. under the inexact match scheme: macro-F1 score: 94.56% and micro-F1 score: 96.78%
Yu et al, 2021^34^	BERT and RoBERTa models	Clinical notes from UF Health IDR	Extraction of SBDoH concepts from clinical narratives for lung cancer screening	Total cohort size: 864 lung cancer patients with 161 933 clinical notes. Randomly selected 500 for training and testing Training: 400 notes, Test: 100 notes	BERT_general strict/lenient F1 score: 0.88/0.90
Lian et al, 2023^35^	BERT-based model, augmented with GPT-2 and sequence pairing	Interviews of thyroid cancer patients from a clinical trial at UW Carbone Cancer Center	Text classification to predict HRQOL trajectory using BERT and GPT-2	100 interviews Training: 70%, Test: 20%, Validation: 10%	AUC of 76.3% for classification of HRQOL accuracy measured by PCS
Nishioka et al, 2022^36^	BERT-based NLP model. Used pre-trained Japanese BERT model released by Tohoku University. MeCab for tokenization for BERT.	Life Palette blog articles, sentences with HFS-related terms	Sentence classification and user identification for detecting HFS symptoms	5492 hand-foot-related sentences, where 149 were HFS-positive. 4:1 train-evaluate ratio	BERT: F1 0.71 in user identification task; F1 0.54 in sentence task
Karlsson et al, 2021^37^	ULMFiT and Google BERT, pre-trained in Finnish	EHRs from Turku University Hospital, Finland; include medical narratives, ICD-10 codes, histology, cancer treatment records, and death certificates	Classification of smoking status (text classification task) to link to cancer outcomes; Train models to classify never, former, and current smokers.	Total number of patients: 29 823 Training model on 5000 tobacco smoking-related phrases and sentences; Use the trained model to analyse 162 000 sentences.	ULMFiT precision: 87.4%, Google BERT precision: 88.2%. ULMFiT (specificity/sensitivity): 96%/96%, 98%/68%, 88%/99%; Google BERT: 96%/96%, 96%/73%, 90%/97% for never, former, current smokers' specificity/sensitivity.
Chen et al, 2023^38^	Fine-tuned PubMedBERT, BioBERT, ClinicalBERT, BioLinkBERT	Clinical notes from lung and esophageal cancer patients undergoing RT at Brigham and Women’s Hospital/Dana-Farber Cancer Institute.	Task 1: Binary classification of esophagitis presence (none vs grades 1-3). Task 2. Binary classification of clinically significant esophagitis (grade ≤1 vs >1). Task 3. Multiclass classification of esophagitis grades (none vs grade 1 vs grade 2-3)	Gold-labeled: 1524 notes, 124 patients; silver-labeled: 2420 notes, 1832 patients. train/test/validation split: 80:10:10	Fine-tuned PubMedBERT performed best. Macro-F1: Task 1—0.92, Task 2—0.82, Task 3—0.74 (lung cancer); Macro-F1: Task 1—0.73, Task 2—0.74, Task 3—0.65 (esophageal cancer)
Solarte-Pabón et al, 2023^39^	BERT, RoBERTa, BETO, Multilingual BERT, RoBERTa Biomedical, RoBERTa BNE	Clinical notes from breast cancer patients at a public hospital in Madrid, Spain	Clinical NER: Extracting named entities from breast cancer clinical notes in Spanish	500 notes, 10-fold cross-validation	Best F score: RoBERTa Biomedical—95.01%
Liu et al, 2023^40^	Bi-LSTM_simple, Bi-LSTM_dropout, BERT	CT and PET/CT report from a tertiary care cancer hospital in India; external validation using MIMIC-III Clinical Database	Classification of radiology reports for lung carcinoma	4064 reports. 3902 for model development and 162 for internal validation	Internal validation F1 scores: Without oversampling: Bi-LSTM_simple 0.89, Bi-LSTM_dropout 0.90, BERT 0.86. With oversampling: Bi-LSTM_simple 0.94, Bi-LSTM_dropout 0.94, BERT 0.90. External validation F1 scores: Without oversampling: Bi-LSTM_simple 0.63, Bi-LSTM_dropout 0.77, BERT 0.80. With oversampling: Bi-LSTM_simple 0.72, Bi-LSTM_dropout 0.78, BERT 0.77.
Bitterman et al, 2023^41^	BioClinicalBERT, RoBERTa	Clinician notes, NAACCR cancer abstracts, and RT prescriptions from HemOnc.org	NER: fine-tuned BioClinicalBERTand RoBERTa for each entity type. Relation Extraction: RoBERTa	495 documents Training: 282, Development: 102, Test: 111; RT event properties: 7981; Relation instances: 12727	NER F1 scores: dose 0.96, fraction frequency 0.88, fraction number 0.94, date 0.88, treatment site 0.67, boost 0.94. Relation extraction: model-averaged 0.86 F1 with gold-labeled entities; end-to-end system 0.81 F1, excelling on NAACCR abstracts (average F1 0.90).
Watanabe et al, 2022^42^	BERT (Japanese BERT model of the Inui and Suzuki Laboratory, Tohoku University)	Blog posts from breast cancer patients in Japan	Multilabel Classification: Extracting and classifying multiple worries from breast cancer patient blogs	2272 blog posts (5-fold cross-validation)	Best Model—Physical (BERT): Precision 0.82; Worst Model—Family/Friends (BERT): Precision 0.58
Liu et al, 2022^43^	MetBERT (fine-tuned on MIMIC-III using BERT base, ClinicalBERT, BioBERT, BlueBERT, PubmedBERT)	Training: MIMIC-III discharge summaries; Testing: Epic system of Spectrum Health	Sequence Classification: Predict metastatic cancer status from EHR clinical notes	Training: 1610 discharge summaries of 4000 patients (178 positively labeled as advanced cancer); Testing: 5024 summaries from 1478 patients	Training: F1 score 0.80 (PubmedBERT); Testing: AUC 0.94 (Spectrum Health)
Mithun et al, 2023^44^	Tabular LASSO, Language LASSO, Fusion LASSO, Language BERT, Fusion BERT	MIMIC-III EHR dataset, clinical notes from Epic Systems Corp. from a Comprehensive Cancer Centre	Text classification: Using clinical notes for predicting ACU in oncology patients post-chemotherapy	The study cohort included 6938 patients. 80% training, 20% testing	Best AUROC: Fusion LASSO (0.778); Best AUPRC: Tabular LASSO (0.411)
Li et al, 2023^45^	BERT (Early Fusion and Late Fusion models), XGBoost	Clinical notes and structured laboratory data from CRC patients admitted for surgery	Text classification: Predicting LM in postoperative CRC patients	1463 CRC patients (split not explicitly specified)	Accuracy: 80.8%, precision of 80.3%, recall of 80.5%, and an F1 score of 80.8% in predicting LM.
Li et al, 2023^46^	CancerGPT (attention-based 2-layer transformer architecture, ∼124M parameters), BERT	DrugComb Portal; Rare tissues	Text-based inference for predicting drug pair synergy in rare tissues	Rare tissues: pancreas (*n* = 39), endometrium (*n* = 68), liver (*n* = 213), soft tissues (*n* = 352), stomach (*n* = 1190), urinary tract (*n* = 2458), bone (*n* = 3985) Various few-shot learning scenarios (0-128 shots) for 7 rare tissues	For pancreas (0 shots): AUPRC (CancerGPT: 0.033), AUROC (GPT-3: 0.789); for endometrium (2 shots): AUPRC (CancerGPT: 0.693), AUROC (GPT-3: 1.00); for liver (64 shots): AUPRC (CancerGPT: 0.782), AUROC (GPT-2: 0.679)
Tan et al, 2023^47^	GatorTron transformer model, alongside other models including transformer variants, Bi-LSTM, and CNN.	CT reports from NCCS; various cancer types	Text classification: Inferring cancer disease response from free-text radiology reports	10 602 reports Training 80%, Development 10%, Test 10%	GatorTron: Accuracy of 0.8916 on test set, 0.8919 on RECIST validation set, improved to 0.8976 with data augmentation.
Zitu et al, 2023^48^	SVM, CNN, BiLSTM, BERT, ClinicalBERT	ICI-OSU corpus from the Ohio State University Cancer Medical Center, including clinical and discharge notes.	Text classification: Detecting ADEs from clinical narratives	1394 clinical notes; n2c2 Shared Task corpus: 505 discharge summaries (MIMIC-III) Train on ICI-OSU: 1394 notes and test on n2c2: 505 summaries and vice versa	ClinicalBERT F score: 0.78 (trained on ICI-OSU, tested on n2c2), 0.74 (trained on n2c2, tested on ICI-OSU); Other methods: F scores 0.55-0.73
Kim et al, 2024^49^	ClinicalBERT, BERT	Data were obtained from Yonsei Cancer Center in South Korea and Yale New Haven Hospital in the United States. The dataset comprised 12 255 CT reports from 2677 patients for training, 3058 CT reports from 670 patients for internal testing, and 1947 CT reports from 273 patients for external testing.	NLP to predict OS from serial CT reports.	The training set included 2677 patients with 12 255 CT reports. The internal testing set consisted of 670 patients with 3058 CT reports, while the external testing set had 273 patients with 1947 CT reports from Yale New Haven Hospital.	The ClinicalBERT model achieved a c-index of 0.811 when trained on up to 15 serial CT reports, compared to 0.653 for a single report. It reached an AUROC of 0.911 for predicting 1-year survival with 15 reports and 0.888 on the external test set. The model showed strong generalizability, with high correlations between predicted survival indices and actual survival times (Spearman’s *r*s = −0.79 for internal testing and *r*s = −0.81 for external testing).
Tay et al, 2024^50^	RadBERT, BioBERT, GatorTron-base, GatorTron-medium	The dataset included 4522 CT reports from 550 patients with 14 types of cancer, collected from the National Cancer Centre Singapore and Singapore General Hospital between May 2000 and February 2022.	Multilabel classification to infer metastatic sites from radiology reports across multiple primary cancers.	Training/Validation: 90% of the dataset was used for training with a 10% development set. Test set: 10% of the dataset was used for testing. External validation was conducted on 3 separate datasets, including 85, 70, and 73 patients with 491 CT reports, 100 PET-CT reports, and 100 MRI reports.	The IE system achieved an F1 score of 0.93 on the test set and 0.89 on the molecular tumour board validation set. GatorTron-Medium performed best on PET-CT and MRI external validation sets, with F1 scores of 0.86 for both. F1 scores for individual cancer types ranged from 0.89 to 0.96.
Rajaganapathy et al, 2024^51^	LLAMA-2, BERT, and GPT-2	Mayo Clinic’s Enterprise Data Warehouse (UDP). The dataset comprised 7884 cancer pathology reports for 7326 unique patients.	Automating the generation of synoptic reports by extracting key data elements from narrative pathology reports using LLMs.	Training: 80% of the data (reports) were used for training. Test: 20% of the data were used for testing, ensuring no patient overlap between the sets.	BERT F1 scores ranged from 0.68 to 1.00. Fine-tuned LLAMA-2 achieved a median F1 of 1 for some elements, outperforming pre-trained models. Accuracy improved from 69% to 81% for shorter reports, with a Pearson correlation of 0.64 between BERT F1 and accuracy.
Zeinali et al, 2024^52^	Symptom-BERT, Bio-BERT, PubMed-BERT, SciBERT, Span-BERT, and Distil-BERT.	Data from the EDW4R at the University of Iowa included 1 million unlabeled cancer patient notes for pre-training, 1112 annotated notes for training/testing, and 180 synthetic ChatGPT-4 notes for validation.	Multi-label classification of cancer symptoms from clinical notes, focusing on 13 symptom groups	The training set used 80% of the 1112 gold-standard annotated notes, while the test set included the remaining 20% along with 180 synthetic notes generated by ChatGPT-4.	Internal validation: Symptom-BERT achieved a micro-averaged F1 score of 0.933 and an AUC of 0.929 across all symptoms. External validation: It achieved an F1 score of 0.831 and an AUC of 0.834 on the synthetic dataset. Symptom-BERT outperformed 7 other BERT variants in both validations.
Nakai et al, 2024^53^	Bert	Clinical notes from 23 225 prostate MRI patients (2017-2022) were analysed for cancer risk factors, pre-MRI pathology, and treatment history.	Classifying sentences from clinical notes to extract relevant prostate cancer-related information, such as family history of cancer, pathology results, and treatment history.	For sentence-level training, 85% of unique sentences were used for training and 15% for testing. The patient-level test set included clinical notes from 603 patients to assess performance.	Sentence-level AUCs ranged from 0.94 to 0.99, with family history scoring highest and treatment history lowest. Outperformed radiologists in sensitivity for family history and prostate nodule extraction but was less accurate for pre-MRI pathology and treatment history classification.
Zhao et al, 2024^54^	BERT, GloVe	Data from 4 medical (MSM) and 5 health/lifestyle (CSM) WeChat accounts, focusing on cancer prevention and treatment. The dataset included 60 843 posts, with 8427 related to cancer.	Multilabel classification of cancer-related posts.	4479 cancer-related articles from MSM and 3948 from CSM. Of these, 35.52% (2993) included prevention information, and 44.43% (3744) covered treatment information.	F1 scores above 85 for prevention and treatment categories in both MSM and CSM. Dense categories (over 10% occurrence) had F1 scores of at least 70, while sparse categories (under 10%) reached F1 scores of at least 50.
Luo et al, 2024^55^	GPT-2 BioGPT PMC-LLaMA	Clinical notes from 3 institutions: Mayo Clinic, EUH, Stanford University.	Extraction of patient-centred outcomes related to breast cancer treatment (eg, side effects like fatigue and nausea) from clinical notes.	Trained on data from the Mayo Clinic (3924 notes), testing on Emory University Hospital (474 notes), and Stanford University (525 notes).	GPT-2 performed overall better with AUC score: Mayo Clinic—0.97 for fatigue and 0.99 for depression. EUH—1.0 for lymphedema, 0.97 for nausea. Stanford—0.95 for anxiety.
Yang et al, 2023^56^	BioBERT, ClinicalBERT, PubMedBERT, BlueBERT, SciBERT, ClinicalTrialBERT	From ClinicalTrials.gov	Text classification to identify 7 exclusion criteria in clinical trial eligibility descriptions.	764 trials in total, with a 5-fold cross-validation. The data was split at the trial level to prevent data leakage.	Performance was evaluated both at the criterion level and the trial level. Across different models, the F1 scores ranged from 0.83 to 1.0 across the 7 exclusion criteria.

This table provides a brief overview of each study's primary researcher, the specific LLM and its application, the nature and origin of the data utilized, the NLP tasks and objectives, the scale of the datasets, and the key metrics used for evaluating model performance. This format offers a comprehensive view of how LLMs are applied quantitatively in cancer research.

Abbreviations: LLM = large language models; NLP = natural language processing; BERT = bidirectional encoder representations from transformers; BiLSTM = bidirectional long short-term memory; NER = named entity recognition; SBDoH = social and behavioral determinants of health; GPT = generative pretrained transformer; HRQOL = health-related quality of life; AUC = area under the curve; PCS = physical component summary; HFS = hand-foot syndrome; EHRs = electronic health records; ICD-10 = International Classification of Diseases-10; RT = radiation therapy; NAACCR = North American Association of Central Cancer Registries; ACU = acute care use; LM = liver metastasis; OS = overall survival; IE = information extraction; EUH = Emory University Hospital; BTN = BiLSTM-transformer network; BERT-WWM = whole word masking version of BERT; RoBERTa = robustly optimized BERT pretraining approach; UF = university of florida; IDR = integrated data repository; UW = university of wisconsin; ULMFiT = universal language model fine-tuning; BETO = spanish BERT; MIMIC-III = medical information mart for intensive care - III; LASSO = least absolute shrinkage and selection operator; AUPRC = area under the precision-recall curve; CRC = colorectal cancer; AUROC = area under the receiver operating characteristic curve; CNN = convolutional neural network; SVM = support vector machine; ADEs = adverse drug events; ICI-OSU = immune checkpoint inhibitor - ohio state university; UDP = unified data platform; MSM = medical social media; CSM = common social media.

**Table 3.** Analysis of LLM chatbots in cancer research.

Author and year	Data source	Chatbot with version	Study objective and prompt formulation	Model efficacy and accuracy	Comparative performance and contextual evaluation
Lyu et al, 2023^57^	Radiology reports: 62 chest CT lung cancer scans, 76 brain MRI metastases scans from the Atrium Health Wake Forest Baptist clinical database	GPT-3 and GPT-4	Objective: To translate radiology reports into plain language for education of patients and healthcare providers. Prompts: translate the report into plain language, provide patient suggestions, provide healthcare provider suggestions.	Average score: 4.268/5. Instances of missing information: 0.080/report. Incorrect information: 0.065/report.	ChatGPT (original prompt): 55.2% accuracy. ChatGPT (optimized prompt): 77.2%. GPT-4 (original prompt): 73.6%. GPT-4 (optimized prompt): 96.8%. Human verification: Two radiologists’ evaluations included focusing on completeness, correctness, and overall score.
Holmes et al, 2023^58^	Radiation oncology physics 100-question multiple-choice examination developed by an experienced medical physicist.	ChatGPT (GPT-3.5, GPT-4), Bard (LaMDA), BLOOMZ	Objective: Evaluate LLMs in answering specialized radiation oncology physics questions. Prompts: ChatGPT (GPT-4) was specifically tested with 2 approaches: explaining before answering and a novel approach to evaluate deductive reasoning by altering answer choices. Performance was compared individually and in a majority vote analysis.	ChatGPT GPT-4: Achieved a 67% accuracy rate in question responses with a stable 14% error rate in each trial. Displayed the highest accuracy among tested models, particularly effective when prompted for explanations before responding. Consistently high performance and notable deductive reasoning skills observed across multiple trials.	ChatGPT surpassed other LLMs as well as medical physicists. Yet, in a majority vote scenario, a collective of medical physicists demonstrated superior performance compared to ChatGPT GPT-4.
Rao et al, 2023^59^	Breast cancer screening and breast pain cases (ACR Appropriateness Criteria); Data size not specified	ChatGPT (GPT-3.5 and GPT-4)	Objective: Assess ChatGPT for radiologic decision support in breast cancer screening and breast pain. Prompts: OE: Determine the single most appropriate imaging procedure. SATA: Concisely assess the listed procedures’ appropriateness. Additional characteristics: Specificity: Yes Iterative prompting: No Context Provided: No Setting boundaries: No	OE for Breast Cancer Screening: Both ChatGPT-3.5 and ChatGPT-4: Average score of 1.830/2. SATA for Breast Cancer Screening: ChatGPT-3.5: 88.9% accuracy. ChatGPT-4: 98.4% accuracy. OE and SATA for Breast Pain: ChatGPT-3.5: OE score of 1.125/2, SATA accuracy of 58.3%. ChatGPT-4: OE score of 1.666/2, SATA accuracy of 77.7%.	ChatGPT-4 significantly improved over ChatGPT-3.5 in decision-making accuracy for both clinical scenarios.
Sorin et al, 2023^60^	10 consecutive early breast cancer cases from MDT discussions, January 2023, at clinic.	ChatGPT 3.5	Objective: Assess ChatGPT in therapy planning for early breast cancer in a MDT setting. Prompts: Inquire about the appropriate treatment approach for a patient with specific details regarding their breast cancer diagnosis. This includes age, TNM status, receptor expressions, HER2 status, Ki67 levels, tumour grading, and any relevant genetic mutations. Additional characteristics: Specificity: Yes Iterative prompting: No Context provided: Yes Setting boundaries: No Directiveness: No	ChatGPT’s recommendations achieved a 16.05% alignment with the MDT, scoring an average of 64.2 out of 400 with a congruence range from 0 to 400.	ChatGPT predominantly offered general treatment modalities and accurately identified risk factors for hereditary breast cancer. However, it sometimes provided incorrect therapy recommendations. Its responses were benchmarked against the MDT recommendations to calculate a clinical score of agreement for determining the level of concordance.
Grünebaum et al, 2023^61^	14 questions about obstetrics and gynaecology, conceived by 4 physicians	ChatGPT 3.5	Objective: Evaluate ChatGPT’s responses to obstetrics and gynaecology questions. Prompts: Specific and context-provided questions without iterative prompting or boundaries.	No numerical score; qualitative comments about ChatGPT’s answers, evaluating the accuracy and relevance of responses.	No direct performance comparison with other models or human experts. ChatGPT’s responses were nuanced and informed but showed potential limitations due to outdated data.
Yeo et al, 2023^62^	164 questions about cirrhosis and HCC	ChatGPT	Objective: Assess the accuracy and reproducibility of ChatGPT in answering cirrhosis and HCC-related questions. Prompts: Evaluated ChatGPT’s responses to 164 diverse questions, independently graded by 2 transplant hepatologists and resolved by a third reviewer. The questions encompassed various aspects of cirrhosis and HCC.	High accuracy in basic knowledge, lifestyle, and treatment. 76.9% of questions answered correctly. However, it failed to specify decision-making cut-offs and treatment durations.	ChatGPT lacked knowledge of regional guidelines, such as HCC screening criteria, compared to physicians or trainees.
Zhu et al, 2023^63^	22 prostate cancer questions based on CDC and UpToDate guidelines; clinical experience of authors	ChatGPT-3.5, ChatGPT 4, and other LLMs	Objective: Assess the performance of ChatGPT and other LLMs in answering prostate cancer-related questions. Prompts: 22 questions covering screening, prevention, treatment, and postoperative complications. The formulation of these questions was intended to test the models’ capacity to provide accurate and comprehensive responses in a medical context.	Most LLMs achieved over 90% accuracy, except NeevaAI and Chatsonic. The free version of ChatGPT slightly outperformed the paid version. LLMs were generally comprehensive and readable.	No direct comparison with human experts, but ChatGPT (with slightly better performance by ChatGPT 3.5) showed the highest accuracy among the LLMs
Sorin et al, 2023^64^	Clinical information of 10 consecutive patients from a breast tumour board	ChatGPT-3.5	Objective: Evaluate ChatGPT-3.5 as a decision-support tool for breast tumour board decisions. Test the chatbot’s ability to process complex medical data and offer recommendations comparable to those of a professional tumour board. Prompts: clinical information of 10 consecutive patients presented at a breast tumour board was input into ChatGPT-3.5. Each patient’s case was detailed, and ChatGPT-3.5 was asked to provide management recommendations.	70% of ChatGPT’s recommendations aligned with tumour board decisions. Moderate to high agreement in grading scores. Grading scores for summarization, recommendation, and explanation varied, with mean scores indicating moderate to high agreement.	No direct comparison with other models or human experts, but ChatGPT’s recommendations aligned closely with those of the tumour board in a majority of cases.
Chen et al, 2023^65^	104 prompts on breast, prostate, and lung cancer based on NCCN guidelines	ChatGPT (gpt-3.5-turbo-0301)	Objective: Evaluate ChatGPT’s performance in providing cancer treatment recommendations aligned with NCCN guidelines. Aimed to explore how differences in query formulation affect ChatGPT’s responses. Prompts: Four zero-shot prompt templates were developed for 26 unique diagnosis descriptions (cancer types ± extent of disease modifiers relevant for each cancer) for a total of 104 prompts.	ChatGPT provided at least 1 NCCN-concordant recommendation for 102 out of 104 prompts (98%). However, 34.3% of these prompts also included at least partially non-concordant recommendations. The responses varied based on prompt type.	No direct comparison with other models or human experts, but the study highlighted limitations in ChatGPT’s ability to provide consistently reliable and robust cancer treatment recommendations.
Nakamura et al, 2023^66^	MedTxt-RR-JA dataset, with 135 de-identified CT radiology reports	GPT-3.5 Turbo, GPT-4	Objective: Evaluate feasibility of ChatGPT to automate the assignment of clinical TNM stages from unstructured CT radiology reports, focusing on optimizing prompt design for better performance. Prompt: Prompt was developed, incorporating the TNM classification rule and stepwise instructions. ChatGPT was guided to extract and assign TNM stages, explain its reasoning, and provide the final output in a JSON-like format.	GPT-4 outperformed GPT-3.5 Turbo in TNM staging accuracy, with GPT-4 scoring 52.2% vs 37.8% for the T category, 78.9% vs 68.9% for the N category, and 86.7% vs 67.8% for the M category.	GPT-4 outperformed GPT-3.5 Turbo, with improvements boosted by including the TNM rule. However, struggled with numerical reasoning, particularly in cases where tumour size determined the T category.
Truhn et al, 2024^67^	Two sets of pathology reports: 100 colorectal cancer reports from TCGA. 1882 neuropathology reports of adult-type diffuse gliomas from the UCL	GPT-4	Objective: Evaluate GPT-4’s ability to extract structured data from unstructured pathology reports and assess its accuracy. Prompt: GPT-4 was provided predefined prompts to extract specific information, such as TNM staging and lymph node involvement, from reports. In some instances, GPT-4 was also tasked with proposing structured reporting templates based on unstructured text.	GPT-4 demonstrated high accuracy in extracting data from colorectal cancer reports, achieving 99% accuracy for T-stage, 95% for N-stage, 94% for M-stage, and 98-99% for lymph node data. In neuropathology reports, it also performed exceptionally well, accurately extracting key variables such as the Ki-67 labeling index and ATRX expression with near-perfect precision.	GPT-4 demonstrated high accuracy compared to manual data extraction, significantly reducing time and costs. However, limitations arose with low-quality scans and handwritten annotations, leading to errors in the OCR step.
Sushil et al, 2024^68^	769 breast cancer pathology reports were retrieved from the UCSF clinical data warehouse, dated between January 1, 2012, and March 31, 2021	GPT-4, GPT-3.5, Starling-7B-beta, and ClinicalCamel-70B	Objective: Evaluated zero-shot classification of breast cancer pathology reports, comparing them to supervised machine learning models like random forests, LSTM with attention, and UCSF-BERT. The goal was to assess if LLMs could reduce the need for large-scale data annotations in clinical NLP tasks. Prompt: LLMs were prompted to extract 12 categories of breast cancer pathology information, such as tumour margins, lymph node involvement, and HER2 status, providing single answers in JSON format.	GPT-4 achieved the highest average macro F1 score of 0.86 across all tasks, surpassing the best supervised model (LSTM with attention), which scored 0.75. GPT-3.5 and other open-source models performed significantly worse, with GPT-3.5 scoring 0.55, Starling 0.36, and ClinicalCamel 0.34.	GPT-4 excelled in zero-shot setups, particularly in tasks with high label imbalance, like margin status inference. However, for tasks with sufficient training data, supervised models like LSTM performed comparably. Open-source models, including Starling and ClinicalCamel, struggled to match GPT-4’s performance.
Liang et al, 2024^69^	80 RCC-related clinical questions, provided by urology experts	ChatGPT-3.5 and ChatGPT-4.0, fine-tuned GPT-3.5 Turbo	Objective: Evaluate ChatGPT’s efficacy in answering clinical questions related to renal oncology, focusing on performance improvement through fine-tuning. Prompt: Binary (yes/no) questions were posed to ChatGPT-3.5, ChatGPT-4.0, and a fine-tuned model, with each question repeated 3 times for consistency.	ChatGPT-4.0 outperformed ChatGPT-3.5 with 77.5% accuracy compared to 67.08%. After iterative optimization, the fine-tuned GPT-3.5 Turbo model achieved 93.75% accuracy.	ChatGPT-4.0 showed a statistically significant improvement over ChatGPT-3.5 (*P* < 0.05) in answering clinical questions, though both exhibited occasional response inconsistencies. The fine-tuned model resolved these issues, achieving 100% accuracy after iterative training, underscoring the potential for optimization through domain-specific training.
Marchi et al, 2024^70^	68 hypothetical clinical cases covering various head and neck cancer stages and tumour sites, based on scenarios from the NCCN Guidelines Version 2.2024	ChatGPT-3.5	Objective: Evaluate ChatGPT’s performance in providing therapeutic recommendations for head and neck cancer compared to NCCN Guidelines, assessing its potential as an AI-assisted decision-making tool in oncology. Prompt: ChatGPT was asked about primary treatment, adjuvant treatment, and follow-up recommendations for hypothetical cases based on NCCN scenarios.	Primary treatment: ChatGPT achieved 85.3% accuracy, 100% sensitivity, and an F1 score of 0.92. Adjuvant treatment: It reached 95.59% accuracy, 100% sensitivity, and an F1 score of 0.96. Follow-up recommendations: ChatGPT attained 94.12% accuracy, 100% sensitivity, and an F1 score of 0.94.	ChatGPT showed high sensitivity and accuracy in line with NCCN Guidelines across tumour sites and stages, though minor inaccuracies appeared in primary treatment. While promising as a cancer care tool, challenges remain in handling complex, patient-specific decisions.
Gu et al, 2024^71^	The study used 160 fictitious liver MRI reports created by 3 radiologists and 72 de-identified real liver MRI reports from patients at Samsung Medical Center, Seoul.	GPT-4 (version gpt-4-0314)	Objective: Assess GPT-4’s ability to extract LI-RADS features and categorize liver lesions from multilingual liver MRI reports to automate key radiological feature extraction. Prompt: A 2-step prompt translated, summarized, and extracted LI-RADS features, using Python-based rules to calculate the category. Prompts were iteratively refined based on performance.	Internal Test (fictitious reports): GPT-4 achieved accuracies of 0.97 to 1.00 for major features like size, nonrim arterial phase hyperenhancement, and threshold growth, with F1 scores of 0.95 to 0.98. External Test (real reports): Accuracy ranged from 0.92 to 0.99 for major LI-RADS features, with F1 scores between 0.89 and 0.94. The overall accuracy for determining the LI-RADS category was 0.85 (95% CI: 0.76, 0.93).	GPT-4 performed slightly lower on external tests due to real report complexity, with higher error rates (4.5% vs 1.8% internal). Misinterpretation and index lesion selection errors were identified. Despite this, GPT-4 shows strong potential for automating radiology feature extraction, though improvements are needed in handling complex cases.
Lee et al, 2023^72^	84 thyroid cancer surgical pathology reports from patients who underwent thyroid surgery between 2010 and 2022 at the Icahn School of Medicine at Mount Sinai.	FastChat-T5 (3B-parameter LLM)	Objective: Assess the efficacy of privacy-preserving LLMs for automated extraction of staging and recurrence risk information from thyroid cancer surgical pathology reports while ensuring patient privacy. Prompt: The model was prompted with 12 expert-designed medical questions, aimed at extracting AJCC/TNM staging data, recurrence risk factors, and tumour characteristics.	Concordance rates between the LLM and human reviewers were 88.86% with Reviewer 1 (SD: 7.02%) and 89.56% with Reviewer 2 (SD: 7.20%). The LLM processed all reports in 19.56 min, compared to 206.9 min for Reviewer 1 and 124.04 min for Reviewer 2.	The LLM achieved 100% concordance for simpler tasks like lymphatic invasion and tumour location but dropped to 75% for complex tasks like cervical lymph node presence. It reduced review time significantly, though further prompt engineering is needed for complex extractions.
Kuşcu et al, 2023^73^	154 head and neck cancer-related questions were compiled from various sources, including professional institutions (eg, American Head and Neck Society, National Cancer Institute), patient support groups, and social media.	ChatGPT Plus, based on GPT-4 (March 2023 version)	Objective: Assess the accuracy and reliability of ChatGPT’s responses to HNC questions, focusing on its potential use for patient education and clinical decision support. Prompt: Questions were entered into ChatGPT individually, and responses were evaluated twice for reproducibility. Two head and neck surgeons graded the answers for accuracy, categorizing them as comprehensive/correct, incomplete/partially correct, mixed, or completely inaccurate/irrelevant.	ChatGPT delivered “comprehensive/correct” answers for 86.4% of the questions, “incomplete/partially correct” for 11%, and “mixed” (both accurate and inaccurate) for 2.6%. No “completely inaccurate/irrelevant” answers were reported.	ChatGPT achieved 100% accuracy in cancer prevention responses and 92.6% for diagnostic questions. It also demonstrated strong reproducibility, with 94.1% consistency across repeated queries. While ChatGPT shows promise as a patient education tool and for clinical decision support, further validation and refinement are needed for medical applications.
Gibson et al, 2024^74^	The dataset included 8 commonly asked prostate cancer questions, derived through literature review and Google Trends.	ChatGPT-4	Objective: Evaluate ChatGPT-4’s accuracy, quality, readability, and safety in answering prostate cancer-related questions, aiming to determine its potential as a patient education tool. Prompt: Eight structured prostate cancer questions, like “What are the symptoms of prostate cancer?” and “What are the pros and cons of treatment options?” were posed to ChatGPT-4, with a request for references in each response.	The PEMAT-AI understandability score was 79.44% (SD: 10.44%), and DISCERN-AI rated the responses as “good” with a mean score of 13.88 (SD: 0.93). Readability algorithm, Flesch Reading Ease score of 45.97, and a Gunning Fog Index of 14.55, indicating an 11th-grade reading level. The NLAT-AI assessment gave mean scores above 3.0 for accuracy, safety, appropriateness, actionability, and effectiveness, indicating general reliability in ChatGPT’s responses.	ChatGPT-4’s outputs aligned well with current prostate cancer guidelines and literature, offering higher quality than static web pages. However, limitations included readability challenges and minor hallucinations (2 incorrect references out of 30). The study concluded that while ChatGPT-4 could enhance patient education, improvements in clarity and global applicability are needed.
Huang et al, 2024^75^	Data was sourced from 2 main datasets: 78 valid lung cancer pathology reports from the CDSA for training. 774 valid pathology reports from TCGA for testing, after excluding invalid or duplicate reports.	ChatGPT-3.5-turbo-16k, GPT-4-turbo	Objective: Assess ChatGPT’s ability to extract structured data (eg, tumour staging, histological diagnosis) from free-text clinical notes. Prompt: ChatGPT extracted tumour features (pT), lymph node involvement (pN), overall stage, and diagnosis, following AJCC seventh edition guidelines, outputting results in JSON format.	ChatGPT achieved 87% accuracy for pT, 91% for pN, 76% for overall tumour stage, and 99% for histological diagnosis, with an overall accuracy of 89%.	ChatGPT-3.5-turbo outperformed NER and keyword search algorithms, which had accuracies of 76% and 51%, respectively. A comparison with GPT-4-turbo showed a 5% performance improvement, though GPT-4-turbo was more expensive. The study also highlighted the challenge of “hallucination” in ChatGPT, especially with irregular or incomplete pathology reports.
Huang et al, 2023^76^	The data included the 38th ACR radiation oncology in-training examination (TXIT) with 300 multiple-choice questions and 15 complex clinical cases from the 2022 Red Journal Gray Zone collection.	ChatGPT-3.5 and ChatGPT-4	Objective: Benchmark ChatGPT-4’s performance on the TXIT examination and Gray Zone clinical cases to evaluate the potential of LLMs in medical education and clinical decision-making in radiation oncology. Prompt: For the TXIT examination, the models provided correct answers without justification. For Gray Zone cases, ChatGPT-4 was prompted as an “expert radiation oncologist” to provide diagnoses and treatment plans.	TXIT exam: ChatGPT-4 achieved 78.77% accuracy, outperforming ChatGPT-3.5’s 62.05%. ChatGPT-4 excelled in areas like statistics, CNS and eye, biology, and physics but struggled with bone and soft tissue and gynaecology topics. Gray Zone Cases: ChatGPT-4 provided highly correct and comprehensive responses, earning a correctness score of 3.5 and a comprehensiveness score of 3.1 (out of 4), as rated by clinical experts.	Compared to ChatGPT-3.5, ChatGPT-4 consistently outperformed in both the TXIT examination and clinical case evaluations. For complex Gray Zone cases, ChatGPT-4 offered novel treatment suggestions in 80% of cases, which human experts had not considered. However, 13.3% of its recommendations included hallucinations (plausible but incorrect responses), emphasizing the need for content verification in clinical settings.
Dennstädt et al, 2023^77^	70 radiation oncology multiple-choice questions (clinical, physics, biology) and 25 OE clinical questions, reviewed by 6 radiation oncologists.	GPT-3.5-turbo	Objective: To assess ChatGPT’s ability to answer radiation oncology-specific multiple-choice and OE questions, evaluating its accuracy and usefulness in this specialized field. Prompt: ChatGPT provided the correct letter (A, B, C, or D) for multiple-choice questions, while radiation oncologists rated its OE responses on correctness and usefulness using a Likert scale.	Multiple-choice questions: ChatGPT provided valid answers for 66 of 70 questions (94.3%), with 60.61% correct. It performed best on physics questions (78.57% accuracy), followed by biology (58.33%) and clinical questions (50.0%). OE questions: 12 of 25 answers were rated as “acceptable”, “good”, or “very good” by all 6 radiation oncologists. Correctness scores ranged from 1.50 to 5.00 on a Likert scale, with a mean of 3.49.	ChatGPT performed reasonably well in answering radiation oncology-related questions but struggled with more complex, domain-specific tasks like fractionation calculations. While ChatGPT can generate correct and useful responses, its performance is inconsistent, particularly in specialized medical areas, due to potential “hallucinations” in answers not grounded in solid evidence.
Choi et al, 2023^78^	Data from 2931 breast cancer patients were collected who underwent post-operative radiotherapy between 2020 and 2022 at Seoul National University Hospital. Clinical factors were extracted from surgical pathology and ultrasound reports.	ChatGPT (GPT-3.5-turbo)	Objective: Develop and evaluate LLM prompts for extracting clinical data from breast cancer reports, comparing accuracy, time, and cost to manual methods. Prompt: Twelve prompts targeted factors like tumour size, lymphovascular invasion, and surgery type, extracting and formatting structured and free-text data for analysis.	The LLM method achieved an overall accuracy of 87.7%, with factors like lymphovascular invasion reaching 98.2% accuracy, while neoadjuvant chemotherapy status had lower accuracy at 47.6%. Prompt development took 3.5 h, with 15 min for execution, costing US$95.4, including API fees.	LLM was significantly more time- and cost-efficient than both the full manual and LLM-assisted manual methods. The full manual method took 122.6 h and cost US$909.3, while the LLM method required just 4 h and US$95.4 to complete the same task for 2931 patients.
Rydzewski et al, 2024^79^	2044 oncology multiple-choice questions from American College of Radiology examinations (2013-2021) and a separate validation set of 50 expert-created questions to prevent data leakage.	GPT-3.5, GPT-4, Claude-v1, PaLM 2, LLaMA 1	Objective: Assess LLMs’ accuracy, confidence, and consistency in answering oncology multiple-choice questions to ensure safe clinical use. Prompt: Models answered questions, provided confidence scores (1-4), and explanations, with prompts repeated 3 times to evaluate consistency. GPT-3.5, GPT-4, Claude-v1, PaLM 2, LLaMA 1	GPT-4 achieved the highest accuracy at 68.7% across 3 replicates, outperforming other models. LLaMA 7B, with 25.6% accuracy, performed only slightly better than random guessing. GPT-4 was the only model to surpass the 50th percentile compared to human benchmarks, while other models lagged significantly.	Model performance varied significantly, with GPT-4 and Claude-v1 outperforming others. Accuracy was lower for female-predominant cancers (eg, breast and gynecologic) compared to other cancer types. GPT-4 and Claude-v1 achieved 81.1% and 81.7% accuracy, respectively, when combining high confidence and consistent responses. GPT-4 Turbo and Gemini 1.0 Ultra excelled in the novel validation set, showcasing improvements in newer models.
Lee et al, 2024^72^	The dataset, totaling 1.17 million tokens, was created by integrating prostate cancer guidelines from sources such as the Korean Prostate Society, NCCN, ASCO, and EAU.	ChatGPT 3.5	Objective: AI-based chatbot that can provide accurate and real-time medical information to cancer patients. Prompt: The chatbot used a fixed prompt to ensure consistency in responses, with the temperature parameter set to 0.1 to generate accurate and reliable answers.	The AI-guide bot’s performance was evaluated using Likert scales in 3 categories: comprehensibility, content accuracy, and readability, with a total average score of 90.98 ± 4.02. Comprehensibility scored 28.28 ± 0.38, accuracy 34.17 ± 2.91, and readability 28.53 ± 1.24.	Compared to ChatGPT, the AI-guide bot demonstrated superior performance in comprehensibility and readability. In evaluations by non-medical experts, the AI-guide bot scored significantly higher than ChatGPT, with *P*-values <0.0001 in both categories. This indicates that the AI-guide bot provided clearer and more accurate medical information, while ChatGPT, drawing from a broader dataset, was less specialized.
Mou et al, 2024^80^	Pathology reports from breast cancer patients at the University Hospital Aachen	GPT-4, Mixtral-8 × 7B	Objective: Transform unstructured pathology reports into a structured format for tumour documentation in line with the German Basic Oncology Dataset. Prompt: LLMs were tasked with extracting key data points from pathology reports, including diagnosis and tumour localization, and converting the information into structured formats according to a predefined data model.	Across 27 examinations, GPT-4 achieved near-human correctness (0.95) and higher completeness (0.97). Mixtral-8 × 7B lagged in both (correctness: 0.90, completeness: 0.95), especially on complex features.	GPT-4 outperformed Mixtral-8 × 7B in both correctness and completeness, particularly in identifying complex features like localization and ICD-10 diagnosis. However, GPT-4 raised concerns about privacy and regulatory compliance, making open-source models like Mixtral more suitable for privacy-sensitive environments. The authors recommend using GPT-4 for performance-critical tasks and Mixtral for privacy-focused scenarios.

This table facilitates a comprehensive understanding of the varying approaches in utilizing different chatbots, highlighting the data types and sources employed, specific objectives and prompt formulations for each study, and detailed insights into the efficacy and accuracy of the models. Additionally, it includes comparative analyses, providing context and benchmarking against other models or human experts, thereby offering a holistic view of the advancements and challenges in applying chatbots in cancer research.

Abbreviations: LLM = large language models; GPT = generative pretrained transformer; ACR = American College of Radiology; OE = open-ended; SATA = select all that apply; MDT = multidisciplinary tumour board; TNM = tumour, node, metastasis; HCC = hepatocellular carcinoma; NCCN = National Comprehensive Cancer Network; TCGA = The Cancer Genome Atlas; UCL = University College Hospitals; UCSF = University of California, San Francisco; OCR = optical character recognition; LSTM = long short-term memory; RCC = renal cell carcinoma; HNC = head and neck cancer; CDSA = Cancer Digital Slide Archive; NER = named entity recognition; ACR = American College of Radiology; HER2 = human epidermal growth factor receptor 2; Ki67 = a marker of cell proliferation; CDC = centers for disease control and prevention; LI-RADS = liver imaging reporting and data system; AJCC = american joint committee on cancer; PEMAT-AI = patient education materials assessment tool for AI; DISCERN-AI = a tool to help healthcare consumers and practitioners in evaluating the quality of healthcare treatment information; NLAT-AI = natural language assessment tool for AI; TXIT = in-training exam; CNS = central nervous system; ASCO = american society of clinical oncology; EAU = european association of urology.

**Table 4.** Overview of qualitative research articles on using LLMs, specifically ChatGPT, in various cancer research and care aspects.

Author and year	Article focus	Summary of findings	Limitations and risks	Key themes
Liu et al, 2023^43^	ChatGPT in clinical support and patient care. Literature review in healthcare.	ChatGPT’s role in clinical decisions, documentation, monitoring, and predictive analytics integration.	Data biases, ethical issues, need for updates.	Accuracy, efficiency, ethical/data bias, challenges, predictive analytics, integration challenges.
Waters et al, 2023^81^	ChatGPT in radiation oncology	Streamlines administrative tasks, patient care, and post-visit instructions.	Clinical decision-making limits, PHI protection, HIPAA compliance.	Administrative efficiency, patient care, ethical/compliance considerations, clinical decision-making limits.
Laios et al, 2023^82^	LLM analysis in ovarian cancer.	LLMs process unstructured data; insights into ovarian cancer.	Need for specific models.	Data processing, personalized treatment, ethical/data privacy considerations, LLM research needs.
Faraji et al, 2023^83^	Medical oncology evaluation with ChatGPT.	Assists in cancer diagnosis/screening; potential in radiologic decisions; personalized medicine.	Ethical considerations, regulatory frameworks.	Diagnostic/screening assistance, radiologic decision support, ethical/regulatory considerations.
Fanconi et al, 2023^84^	LLMs potential analysis in oncology.	Enhances clinical decision-making, patient education; processes healthcare texts.	Ethical and data privacy concerns.	Clinical decision-making, patient education, text processing, ethical/privacy concerns.
Lyon et al, 2023^85^	AI evaluation in oncology nursing and writing.	Chatbots assist in literature reviews, research summarization, translations.	Ethical/legal concerns in scholarly AI use.	Scholarly support, ethical/legal AI concerns, oncology nursing role.
Iannantuono et al, 2023^86^	LLMs in cancer care	Provides accurate cancer care information.	Accuracy limitations, data obsolescence, misinformation risk.	Information accuracy, ethical implications, AI’s future in cancer care.
Ramamurthi et al, 2023^87^	LLM (ChatGPT-4) in surgical oncology.	Automating clinical report generation, providing interactive patient support, and aiding in treatment navigation	Data drift, privacy concerns, and ethical challenges associated with the clinical use of LLMs.	AI in surgical oncology, ethics, and privacy, collaborative innovation
Murmu et al, 2024^88^	AI in cancer research	AI models, including NLP and LLMs, are improving cancer diagnosis, treatment prediction, and personalized care	Highlights challenges such as data heterogeneity, reproducibility issues, bias, lack of standardized reporting, privacy concerns, ethical issues, and the black-box nature of AI models	AI for predictive models, imaging analysis and clinical data extraction. Challenges related to data complexity, reporting standards, and ethics. Importance of explainable AI.

This table is designed to give a concise yet comprehensive view of each study, including the article’s focus, a summary of the key findings, identified limitations and risks associated with using LLMs in the given context, and the primary themes explored. This format helps understand the diverse qualitative impacts, challenges, and considerations of using LLMs like ChatGPT in cancer research and patient care.

Abbreviations: LLMs = large language models; AI = artificial intelligence; NLP = natural language processing; PHI = protected health information; HIPAA = Health Insurance Portability and Accountability Act.

### Potentials of LLMs in oncology

#### Advancements of LLMs in cancer research

The adaptability of LLMs in patient care is highlighted by Bitterman et al^41^ and Watanabe et al^42^ focusing on radiation therapy events and analysing patient concerns in breast cancer blogs (see Table 2). Watanabe et al^42^ analysed patient concerns in 2272 breast cancer blog posts to extract and classify multiple worries using the BERT (bidirectional encoder representations from transformers) model, with precision scores ranging from 0.58 to 0.82. These studies suggest the potential of LLMs’ in patient-centred care through deep insights into patient experiences. Additionally, Li et al^45^ and Li et al^46^ showcased notable advancements in LLM architecture (see Table 2). Li et al^45^ combined BERT with XGBoost to predict liver metastases in postoperative colorectal cancer patients, achieving an F1 score of 80.8%, while Li et al^46^ developed CancerGPT to identify drug-pair synergies across 7 rare tissue types, demonstrating strong AUROC (area under the receiver operating characteristic curve) scores, such as 1.00 for endometrium with 2-shot learning. These models offer potential in integrating diverse data, aiding in predicting liver metastases, and identifying drug synergies. Research from Karlsson et al^37^ to Bitterman et al^41^ demonstrated LLMs’ potential for diagnostics and patient interaction (see Table 2). Liu et al^43^ and Waters et al^81^ presented ChatGPT as an effective tool in clinical decision support and radiation oncology (see Table 4). Furthermore, Tan et al,^47^ Tay et al,^50^ Kim et al,^49^ and Zitu et al^48^ demonstrated the use of GatorTron and ClinicalBERT for inferring cancer disease response, automating the identification of metastatic sites from radiology reports, survival prediction in pancreatic cancer, and detecting adverse drug events, marking advancements in LLM technology for cancer diagnosis and treatment (see Table 2). Zitu et al^48^ assessed the generalizability of LLMs and achieved an F score of 0.78 to identify adverse drug events from clinical notes. Laios et al^82^ and Faraji et al^83^ explored AI’s potential in ovarian cancer research and its broader implications in oncology (see Table 4). Laios et al^82^ discussed LLMs’ capability to process extensive unstructured data in ovarian cancer, while Rajaganapathy et al^51^ showed use of LLMs such as LLAMA-2 to streamline the process of generating synoptic reports (see Table 2). Faraji et al^83^ extended this discussion to the utility of ChatGPT in cancer diagnosis and radiologic decision-making, emphasizing the importance of ethical deployment and its role in personalized and precision oncology (see Tables 2 and 4).

#### Innovative architectures in LLMs for cancer diagnostics

PathologyBERT, developed by Santos et al,^32^ was a model specifically designed for pathology reports on Emory University Hospital data, demonstrated promising diagnostic accuracy in breast cancer classification. With 347 173 reports used for training, validation, and testing, the model achieved a 15% masked prediction accuracy of 0.73 and an F1 score of 0.70 for classifying non-breast cancer labels (see Table 2). Similarly, Zhang et al^33^ developed a BERT-based BiLSTM-Transformer network, which excelled in extracting clinical entities from 359 Chinese CT reports for lung cancer screening and staging. The model’s performance under the exact match scheme yielded a macro-F1 score of 85.96% and a micro-F1 score of 90.67%, highlighting its potential for enhancing clinical decision-making (see Table 2). Additionally, Zeinali et al^52^ developed specialized models like Symptom-BERT, demonstrating high efficacy in detecting cancer symptoms in clinical notes with a micro-averaged F1 score of 0.933.

#### Personalized cancer care and patient-centred approaches

Lian et al^35^ augmented a BERT model with GPT-2, targeting the prediction of health-related quality of life (HRQOL) trajectories in thyroid cancer patients using 100 interview transcripts from a clinical trial at the UW Carbone Cancer Center. The model achieved an area under the curve of 76.3% for predicting HRQOL accuracy, highlighting the trend towards more personalized and patient-centred approaches in cancer care (see Table 2). Complementing this, Nishioka et al^36^ employed BERT models to detect hand-foot syndrome (HFS) symptoms in 5492 blog entries related to cancer, achieving an F1 score of 0.71 for user identification and 0.54 for sentence classification. Zhao et al^54^ analysed data from WeChat cancer-related posts using LLMs to explore patient-generated data, with F1 scores above 85 for prevention and treatment categories (see Table 2).

#### Diverse applications and management in cancer research

The range of LLM applications is illustrated by studies such as Karlsson et al^37^ who used ULMFiT and BERT for analysing smoking status with good accuracy in electronic health records (EHRs) and Chen et al^38^ who focused on classifying esophagitis severity. Yang et al^56^ explored the use of LLMs in automating clinical trial eligibility criteria, while Mou et al^80^ integrated LLMs into hospital data management systems to improve cancer diagnosis (see Tables 2 and 3). These studies demonstrate the versatility of LLMs in handling diverse data types, such as clinical notes and pathology reports, and their capacity to contribute to different aspects of cancer management, from lifestyle factor analysis to the management of radiotherapy-induced toxicities.

#### Medical communication and radiological decision support

Lyu et al,^57^ Rao et al,^59^ and Nakamura et al^66^ demonstrated feasibility of ChatGPT in medical communication and radiological decision support (see Table 3). Lyu et al^57^ utilized ChatGPT to translate complex radiology reports into plain language, improving understanding for both patients and healthcare providers. The translations achieved high accuracy, with notable improvements noted in GPT-4 over GPT-3. Meanwhile, Rao et al,^59^ Luo et al,^55^ Nakamura et al^66^ assessed ChatGPT and other GPT models’ efficacy in automatic TNM (tumour, node, metastasis) staging, radiological decision-making, particularly in breast cancer screening, breast pain, and adverse event cases, where GPT-4 demonstrated notable accuracy and the potential to improve clinical workflow (see Table 2). For instance, ChatGPT-4 achieved 98.4% accuracy in breast cancer screening decisions, surpassing ChatGPT-3.5’s 88.9%.^59^

#### Performance in specialized medical fields

The studies by Holmes et al,^58^ Grünebaum et al,^61^ and others emphasized ChatGPT’s capabilities in specialized medical fields (see Table 3). Holmes et al^58^ focused on radiation oncology physics, demonstrating ChatGPT’s high accuracy and consistency in responding to complex questions. The authors suggest that while ChatGPT shows potential in specialized knowledge areas, its role may be best suited as a knowledgeable assistant, requiring careful integration into the clinical workflow to complement human expertise.

#### Prompt engineering and processing in ChatGPT applications

Studies by Lyu et al,^57^ Grünebaum et al,^61^ Zhu et al,^63^ and Chen et al^65^ highlight the importance of prompt formulation in ChatGPT’s response accuracy and relevance (see Table 3). Lyu et al^57^ reported improved accuracy in radiology report translations with well-crafted prompts, while Grünebaum et al^61^ found that prompt specificity significantly influenced ChatGPT’s responses in obstetrics and gynaecology queries. The work of Zhu et al^63^ on prostate cancer-related questions and Chen et al^65^ on the assessment of cancer treatment recommendations according to NCCN (National Comprehensive Cancer Network) guidelines demonstrated how prompt variations affect ChatGPT’s output quality. Collectively, these studies emphasize the necessity of meticulous prompt engineering for precise and relevant ChatGPT responses in complex medical contexts.

Additionally, Dennstädt et al,^77^ Kuşcu et al,^73^ and Truhn et al^67^ show that ChatGPT and LLMs have the potential to access real-time, personalized medical information for cancer patients, improving patient engagement and decision-making. Gu et al^71^ highlight GPT-4’s ability to synthesize large datasets for personalized oncology treatment recommendations. Truhn et al^67^ emphasize GPT-4’s role in extracting structured data from unstructured pathology reports, reducing the workload for human experts. Sushil et al^68^ demonstrate the use of LLMs like RadBERT and ClinicalBERT to automate metastatic site identification in radiology reports, highlighting their scalability in cancer research. Rydzewski et al^79^ further highlight GPT-4’s performance on oncology-specific multiple-choice questions, where it achieved an accuracy of 68.7%, outperforming other models.

#### Risks (challenges, ethical, privacy)

Incorporating LLMs like ChatGPT in cancer research and healthcare can be met with several risks where technical limitations intersect with ethical and practical challenges. Studies by Lyu et al^57^ and Iannantuono et al,^86^ for instance, described inconsistencies in LLMs and their reliance on potentially outdated information, raising concerns on their ability to provide current and reliable data (see Tables 3 and 4). Holmes et al^58^ and Liu et al^43^ highlight risks such as misdiagnosis and data biases in complex medical scenarios, as well as the ethical implications of misinformation (see Tables 3 and 4). While Liu et al^43^ and Waters et al^81^ discussed LLMs’ potential in clinical decision-making and efficiency in administrative tasks, they also cautioned against over-reliance on these AI models, emphasizing the need for human oversight, especially in safeguarding patient data privacy and adhering to healthcare regulations like the Health Insurance Portability and Accountability Act (HIPAA; see Table 4).

Laios et al^82^ and Faraji et al^83^ addressed the limitations of LLMs, such as their challenges in providing accurate cancer care information and the necessity for continuous updates to avoid data obsolescence (see Table 4). These studies underscore the need for expert verification to prevent the spread of misinformation and the potential ethical implications of using LLMs in cancer care, particularly regarding the quality and reliability of information provided. Additionally, Sorin et al,^64^ Lyon et al,^85^ and Iannantuono et al^86^ explored the broader ethical, privacy, and future implications of LLMs in oncology, focusing on their roles in clinical decision-making, patient education, and oncology nursing (see Tables 3 and 4). Iannantuono et al,^86^ Lee et al^72^ further ask for an accurate oncology expert-driven verification to avoid any potential error. They highlight the challenges of integrating AI tools like ChatGPT in academic and clinical settings, where issues such as intellectual property rights and human subjects’ protection are present.

Dennstädt et al^77^ and Huang et al^76^ pointed out the risk of “hallucinations” in LLMs, where the models produce plausible but incorrect information. Kuşcu et al^73^ and Nakamura et al^66^ (see Table 3). also found that hallucinations can result in inaccuracy, which could lead to improper treatment recommendations. Gu et al^71^ and Lee et al^72^ discussed the overconfidence exhibited by LLMs in their outputs, where the models presented incorrect information with high certainty (see Table 3). Additionally, Lee et al^72^ and Truhn et al^67^ noted that LLMs often struggle with complex medical language and report formats, leading to inconsistencies in clinical data interpretation.

Although the capabilities of LLMs in interpreting complex datasets, as illustrated by Chen et al,^65^ can be beneficial, they must be carefully balanced against concerns over data accuracy and ethical considerations, as noted by Yeo et al^62^ and Zhu et al^63^ (see Table 3). These insights emphasize the importance of developing domain-specific LLMs and robust datasets, as suggested by Lian et al^35^ and Nishioka et al,^36^ to cater to the unique requirements of oncology (see Table 2). In summary, these studies highlight the multifaceted risk environment surrounding using LLMs in cancer research and healthcare, warranting the need for informed approaches to integrating LLMs in these fields, ensuring accuracy, compliance with ethical standards, and diligent management of data privacy concerns.

#### Safeguards

Implementing safeguards for using LLMs like ChatGPT in cancer research and healthcare is a multifaceted endeavour, considering their inherent limitations and ethical implications. Drawing from the collective insights of studies ranging from Santos et al^32^ to Holmes et al,^58^ there is a clear emphasis on the need for specialized, domain-specific models that address the unique challenges of oncology (see Tables 2 and 3). These models, as suggested by Iannantuono et al^86^ and Laios et al,^82^ should be designed with a focus on ethical and privacy considerations, ensuring that patient data is handled ethnically and with the utmost care (see Table 3).

The studies by Yeo et al,^62^ Zhu et al,^63^ and Chen et al^65^ advocate for a cautious application of LLMs in cancer research and treatment (see Table 3). They suggest leveraging ChatGPT’s capabilities in patient education and decision support while being aware of its limitations in clinical applications. This cautious approach supports the importance of integrating LLMs into healthcare systems in a way that complements the expertise of medical professionals. It also highlights the importance of continuously evaluating and adapting these models to ensure they remain effective, accurate, and ethically sound.

By establishing clear AI reporting guidelines, Murmu et al^88^ and Ramamurthi et al^87^ emphasize the importance of transparency, flexibility, accuracy, representativeness, and interpretability as essential safeguards for deploying LLMs in the rapidly evolving cancer research domain (see Table 4). Yu et al^34^ illustrate the importance of adaptable models in response to evolving healthcare policies, whereas Luo et al,^55^ Nakai et al,^53^ Zeinali et al,^52^ Choi et al,^78^ and Sushil et al^68^ highlight the importance of fine-tuned model on domain-specific medical tasks, ensuring research data’s continued relevance and accuracy (see Table 2). Complementing this, Solarte-Pabón et al^39^ underscore the role of precise annotations in maintaining the accuracy and reliability of models, especially in complex fields like oncology (see Table 2). Addressing dataset challenges, Liu et al^40^ highlight the downsides of imbalanced datasets, and Mithun et al^44^ showcase the necessity of techniques like dropout regularization in training models on imbalanced datasets, ensuring representative and unbiased model outputs. Gu et al^71^ advocate for the use of retrieval-augmented generation systems, which allow LLMs to access verified external databases. Fanconi et al^84^ highlight the imperative for interpretable models to mitigate risk bias, particularly in sensitive applications like chemotherapy patient care, enhancing model applications’ trustworthiness and clinical utility (see Table 2). Lee et al^72^ and Truhn et al^67^ underscore the value of deploying open-source LLMs within healthcare institutions to safeguard patient privacy and allow for closer supervision during model training and application.

## Discussion

### Recommendation for tool usage in specific situations

The review highlights the diverse applications of LLM tools in oncology settings from the literature, describing their use for interpreting complex medical data. For instance, models such as PathologyBERT^32^ and BERT-based BiLSTM-Transformer networks^33^ are shown to be effective for pathology report interpretation, contributing to diagnostic accuracy in breast and lung cancer classification. For patient education and decision support scenarios, GPT-4 outperforms other models for its advanced language translation and comprehension capabilities, particularly useful for converting complex radiology reports into plain language and aligning treatment suggestions with guidelines, as seen in studies like Marchi et al,^70^ Gibson et al^74^ on head-neck and prostate cancer. Iterative optimization of ChatGPT’s model could further improve its response accuracy in specific clinical scenarios, such as in renal oncology, where improved decision-making support is important.^69^ GPT-4 has shown improved accuracy in automating tasks such as TNM stage assignment from unstructured radiology reports, outperforming previous models in key areas like TNM classification.^66^ The use of ChatGPT in specialized fields like radiation oncology physics has also shown good accuracy and consistency, making it a feasible approach for addressing complex, domain-specific queries. ChatGPT’s role in processing large volumes of clinical notes also demonstrates its feasibility for structured data extraction to support clinical decision-making.^75^ These tailored approaches to tool selection, thus, based on the specific needs of each scenario in cancer research and treatment, optimize the potential of LLM technologies, thereby improving oncology care and research outcomes.

### Planning for risks and safeguards when using LLM tools

Proactive and comprehensive strategies to manage risks and implement safeguards are essential when deploying LLM tools in cancer research. For instance, continuous updates and expert oversight are needed to address data biases and outdated information while also adhering to ethical standards and privacy regulations in handling patient health information.^57^^,^^60^^,^^86^

Future research should also consider a holistic strategy that includes developing specialized AI tools, regular regulatory supervision, unwavering commitment to ethical compliance, and continuous evaluation and improvement. Balancing such approaches with stringent regulatory review, standardized testing, and certification of tools, as well as robust validation processes, is essential for harnessing AI’s transformative power in oncology while ensuring top-tier patient care and data security.^89^ For example, GPT-4 shows effectiveness in radiological feature extraction but struggles with low-quality scans and handwritten annotations, resulting from OCR (optical character recognition) errors, highlighting the need for rigorous validation.^67^ Ethical considerations are pivotal, particularly adherence to data privacy laws and high ethical standards in AI utilization. Collectively, these measures guarantee AI’s responsible, transparent application in healthcare, supported by comprehensive protocols and training for healthcare professionals. Such a framework would assist the integration of tools like ChatGPT into clinical practice, aligning with the highest standards of patient-centred care.

### Suggesting a pathway to adopt LLMs into the clinical workflow

Integrating LLMs into clinical workflows should be a balanced and dynamic process. A pathway to adoption includes developing specialized, domain-specific AI tools that address the unique requirements of oncology. Consistent regulatory oversight and ethical compliance are essential for safe and effective use. The review also suggests that LLMs should complement, not replace, medical expertise to enhance patient education, decision support, and administrative efficiency. Continuous evaluation and adaptation of these models are crucial to maintaining their effectiveness and relevance. By following these guidelines, LLMs can be effectively integrated into clinical settings, enhancing patient care while upholding the highest standards of medical practice.

### Limitations and future works

#### Lack of systematic review guidelines and selection bias

We followed a narrative review approach influenced by the PRISMA framework but did not fully adhere to systematic review guidelines. As a result, our approach may have led to the omission of relevant studies. Additionally, although multiple reviewers were involved, the manual screening process could have human bias in article selection.

#### Limited scope to text-based LLM applications

The focus on text-based LLM applications in cancer research excludes studies using other data types (eg, imaging, omics), limiting the generalizability of our findings. Future reviews should include a broad range of data modalities for a more comprehensive assessment.

#### Potential over-simplification in study classification

Our classification of studies into quantitative methods, chatbot-focused studies, and qualitative discussions may not fully capture the diversity of approaches. Additional categorizations could provide more insights in this regard.

#### Lack of detailed guidelines for ethical implementation

We emphasized the need for ethical safeguards and regulatory oversight. However, this review may lack specific guidelines for responsible LLM implementation. Future research should aim to develop concrete recommendations for the ethical use of AI, especially LLMs in cancer.

## Conclusion

This review of 59 articles on LLMs in cancer research highlights their potential and challenges in oncology. Quantitative studies suggest that LLM may contribute to advancements in diagnostics and patient care, while chatbot-focused studies, particularly on ChatGPT, indicate their potential utility in clinical support and patient communication. Conversely, qualitative analyses reveal concerns about ethics, data privacy, and the need for tailored models. The integration of LLMs in cancer research and healthcare presents a promising avenue for improving patient care. Still, this pathway requires a cautious and balanced approach rooted in continuous evaluation, adherence to ethical standards, and a strong commitment to safeguarding data privacy.
